# AAV‐mediated gene transfer of DNase I in the liver of mice with colorectal cancer reduces liver metastasis and restores local innate and adaptive immune response

**DOI:** 10.1002/1878-0261.12787

**Published:** 2020-09-05

**Authors:** Yujia Xia, Jiayi He, Hongji Zhang, Han Wang, George Tetz, Casey A. Maguire, Yu Wang, Amblessed Onuma, Dmitry Genkin, Victor Tetz, Alexey Stepanov, Stanislav Terekhov, Valeria Ukrainskaya, Hai Huang, Allan Tsung

**Affiliations:** ^1^ Division of Surgical Oncology Department of Surgery The Ohio State University Wexner Medical Center Columbus OH USA; ^2^ Department of Gastroenterology Tongji Medical College Tongji Hospital Huazhong University of Science and Technology Wuhan China; ^3^ Department of Pediatrics Tongji Medical College Tongji Hospital Huazhong University of Science and Technology Wuhan China; ^4^ Human Microbiology Institute New York NY USA; ^5^ CLS‐Therapeutics New York NY USA; ^6^ Molecular Neurogenetics Unit Harvard Medical School Massachusetts General Hospital Charlestown MA USA; ^7^ M.M. Shemyakin and Yu.A. Ovchinnikov Institute of Bioorganic Chemistry Russian Academy of Sciences Moscow Russia

**Keywords:** adeno‐associated virus, Deoxyribonuclease I, metastatic colorectal cancer, neutrophil extracellular traps

## Abstract

Liver metastasis is the main cause of colorectal cancer (CRC)‐related death. Neutrophil extracellular traps (NETs) play important roles in CRC progression. Deoxyribonuclease I (DNase I) has been shown to alter NET function by cleaving DNA strands comprising the NET backbone. Moreover, DNase I displays high antimetastatic activity in multiple tumor models. To circumvent long‐term daily administrations of recombinant DNase I, we have developed an adeno‐associated virus (AAV) gene therapy vector to specifically express DNase I in the liver. In this study, we demonstrate AAV‐mediated DNase I liver gene transfer following a single intravenous injection suppresses the development of liver metastases in a mouse model of CRC liver metastasis. Increased levels of neutrophils and NET formation in tumors are associated with poor prognosis in many patients with advanced cancers. Neutrophil infiltration and NET formation were inhibited in tumor tissues with AAV‐DNase I treatment. This approach restored local immune responses at the tumor site by increasing the percentage of CD8^+^ T cells while keeping CD4^+^ T cells similar between AAV‐DNase I and AAV‐null treatments. Our data suggest that AAV‐mediated DNase I liver gene transfer is a safe and effective modality to inhibit metastasis and represents a novel therapeutic strategy for CRC.

## Graphical abstract

Liver metastasis is a major cause of colorectal cancer‐related death. Although DNase I displays antimetastatic activity by inhibition of NETs, its clinical use is limited due to its short biologic half‐life. AAV‐mediated gene expression of DNase I is a novel therapeutic strategy that could reduce the development of liver metastases through modulation of innate and adaptive tumor immunity.

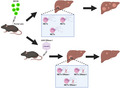

**Abbreviations**
AAV: adeno‐associated virus
CRC: colorectal cancer
DNase I: deoxyribonuclease I
IP: intraperitoneal
IV: intravenous
NETs: neutrophil extracellular traps

## 1 Introduction

Colorectal cancer (CRC) is a leading cause of cancer‐associated death and the third most common cancer worldwide [1]. The liver is the most frequent site of CRC metastasis, as the majority of intestinal mesenteric drainage enters through the hepatic portal venous system [2]. Population‐based studies have shown that ~ 25–30% of patients diagnosed with CRC have synchronous liver metastasis during the course of their disease [3, 4]. Unfortunately, despite recent oncological and surgical advances, only about 25% of CRC patients are amenable to liver resection, which is regarded as the only curative treatment [3, 5]. Once the disease spreads to other organs, benefits of conventional chemotherapy and targeted therapy (e.g., bevacizumab and cetuximab) are limited [6, 7]. Therefore, it is important to develop novel therapeutic strategies for distant metastasis.

Neutrophil extracellular traps (NETs) are composed of extracellular strands of decondensed chromatin expelled from activated neutrophils, having over 15 × 10^3^ base pairs of DNA in length and organized into three‐dimensional web‐like structures [8, 9]. Accumulated evidence suggests that NETs are ‘protagonists’ of cancer progression: Cancer cells provoke the innate immune system to produce more NETs in order to promote their own growth, invasion, and metastasis [10, 11, 12]; increased deposition of NETs within the tumor tissue predicts poor survival [13]; excessive NETs formation trigger coagulopathies and thrombotic events [14], inflammatory states [15], and permanent organ damage to the respiratory, cardiovascular, and renal systems in cancer patients [16]. In patients receiving cytotoxic chemotherapy, NETs facilitate the development of drug resistance [17] and related toxicities [18].

Deoxyribonuclease I (DNase I) is an endonuclease that selectively cleaves the phosphodiester bond in DNA, which is the major structural component of NETs; thus, the DNA scaffold of NETs can be destroyed by DNase I [8]. Single daily intravenous (IV) or intraperitoneal (IP) injections of DNase I display potent antimetastatic activity in multiple animal tumor models [19, 20, 21, 22]. According to our previous work, daily systemic administration of DNase I at the dose of 50 μg·per mouse per·day for 3 weeks provides meaningful suppression of primary tumor growth and metastasis development in subcutaneous and metastatic models of MC38 murine colon adenocarcinoma in C57BL/6 mice as well as in subcutaneous HCT116 human colorectal carcinoma and human Huh7 liver carcinoma models in NU/J mice [23].

However, recombinant DNase I protein has a relatively short half‐life of 3–4 h with serum concentrations displayed variable multicompartment distribution and dose‐proportional profiles after IV injection [24, 25]. Monomeric G‐actin released from neutrophils as the result of NETs formation [26] almost entirely inhibits DNase I enzymatic activity [27]. Considering pharmacokinetic and pharmacodynamics variables, multiple daily injections of DNase I is required to achieve sufficient drug exposure. Bearing in mind the potential lifelong nature of treatment, these factors create a significant challenge for the use of DNase I as a therapeutic agent for cancer treatment in human trials.

Gene therapy is a powerful therapeutic tool that can transduce a range of cells to produce and secrete therapeutic proteins. The adeno‐associated virus (AAV) is a versatile viral vector technology that can be engineered for very specific functionality in gene therapy applications and is proven to be one of the safest gene transfer strategies [28]. AAVs demonstrated safety and efficacy profile in the clinic using liver‐directed gene therapies, and its persistence and long‐term expression in differentiated cells make this vector of choice to explore DNase I gene transfer [29]. Recent efforts to engineer new AAV vectors have shown that transduction efficiency can be greatly improved through capsid engineering approaches. One such example is the engineered AAV capsid, Anc80L65, which has been demonstrated to be a highly potent vehicle for *in vivo* liver targeted gene transfer [30]. To develop an effective and clinically applicable delivery system providing long‐term expression of DNase I in the liver for antitumor therapy, we generated an AAV expressing DNase I transgene. Human DNase I cDNA was put under the control of a liver‐specific promoter, cloned into an AAV expression cassette, and packaged within the Anc80L65 capsid. This study for the first time evaluates the efficacy of AAV‐mediated DNase I liver gene transfer in a mouse model of CRC liver metastasis.

## 2 Materials and methods

### 2.1 Cell culture

Murine cancer cell line of MC38 colon carcinoma (Kerafast, Boston, MA, USA) and human hepatoma cell line HepG2 (American Type Culture Collection, Manassas, VA, USA) were cultured in Dulbecco's modified Eagle's medium (DMEM; Hyclone, Logan, UT, USA) supplemented with 10% FBS (Hyclone) and 1% penicillin/streptomycin (Thermo Fisher Scientific, Waltham, MA, USA) at 37 °C in a 5% CO_2_ incubator (Thermo Fisher Scientific).

### 2.2 Luciferase transfection and bioluminescence imaging

MC38 tumor cells expressing GFP and firefly luciferase (FLuc) genes were generated using FLuc‐F2A‐GFP‐IRES‐Puro Lentivirus (Biosettia, San Diego, CA, USA) then selected with hygromycin (Thermo Fisher Scientific). For imaging, mice were anesthetized with inhaled isoflurane followed by IP injection of potassium luciferin (300 mg·kg^−1^; Gold Biotechnology, St. Louis, MO, USA). After 10 min to allow for luciferin distribution, mice were imaged using the Optical imaging system IVIS Lumina II (PerkinElmer Inc., Shanghai, China), according to the manufacturer's instructions. Analysis of resultant data was performed using living image software (PerkinElmer Inc., Shanghai, China). Regions of interest were manually selected and quantified for average photon flux (photons/second/cm^2^/steradian).

### 2.3 Animals and tumor models

Male and female wild‐type (C57BL/6) mice were purchased from Jackson Laboratories (Bar Harbor, Maine, USA). Animal protocols were approved by the Animal Care and Use Committee of The Ohio State University, and the experiments were performed in adherence to the National Institutes of Health Guidelines. In the liver metastasis models, the anesthetized mice were placed in a supine position. After disinfecting the skin in the area of surgery, a median abdominal incision was performed followed by mobilization of the duodenum to identify the portal vein. One million MC38 cells were injected into the portal vein using a 30‐G needle. After removal of the needle, bleeding was stopped by gently pressing the puncture site with a cotton swab. After injection, the intestine was repositioned and the abdominal wall was closed with nonabsorbable sutures. A dose of 1.05 × 10^12^ GC per mouse vector genomes was systemically administered through the tail vein on 4 days after MC38 injection. Blood was collected before MC38 injection and 21 days after MC38 injection. Livers were harvested at 21 days after MC38 injection.

### 2.4 AAV expression plasmid construction

**Fig. 1:**
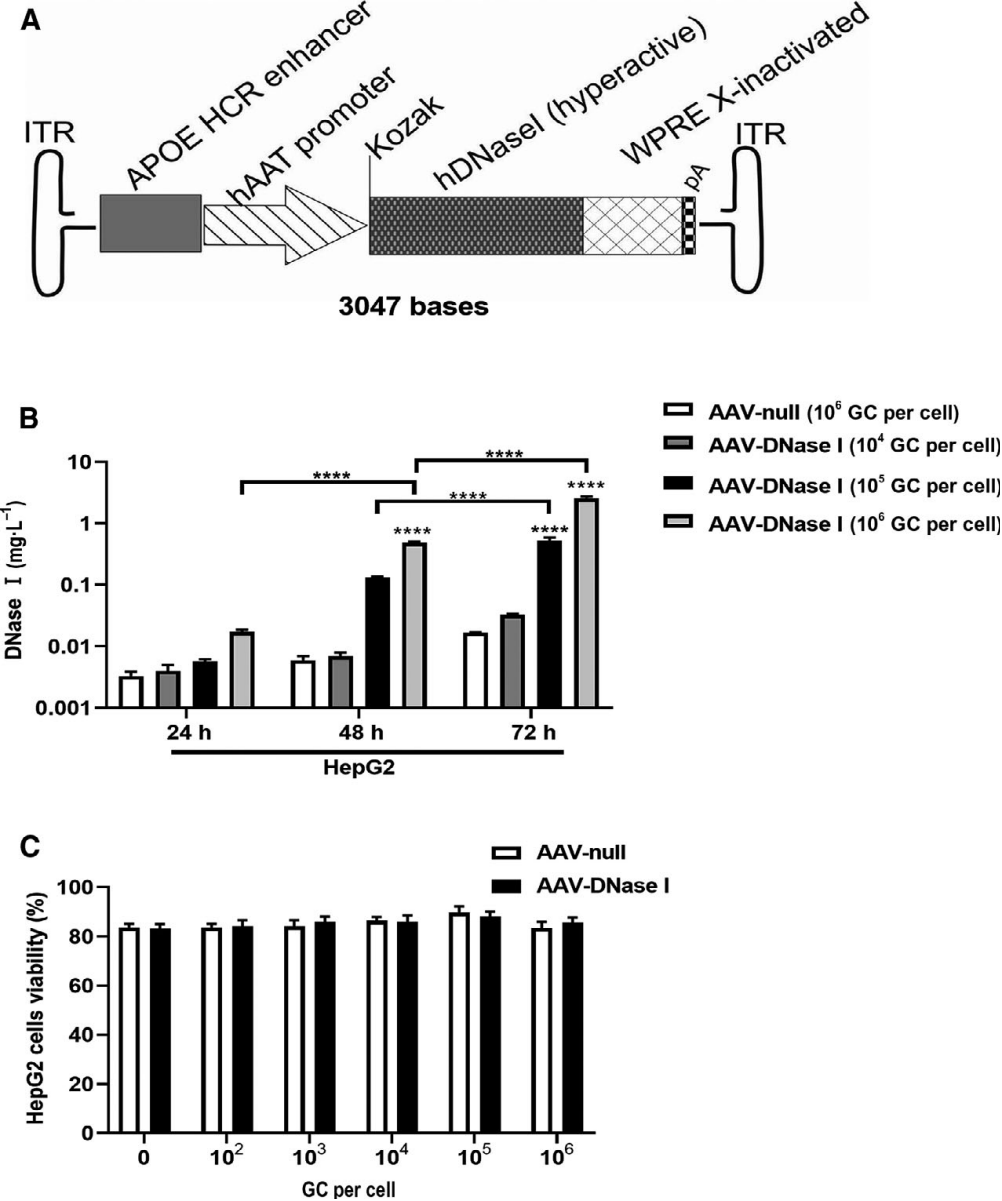
AAV‐DNase I transduction of cultured human hepatoma cells leads to DNase I secretion. (A) The schematic construct of pAAV‐ApoEHCR enhancer‐hAAT promoter‐hDNase I (hyperactive)‐WPRE Xinact (AKA, CLS‐014). hAAT promoter, human alpha antitrypsin promoter. (B) DNase I levels were determined at 24–72 h post‐transduction with different AAV vector concentrations in HepG2 cells. Levels of AAV‐DNase I in culture media changes with time and vector concentration. *n* = 3. (C) Viability of cells as indexed by trypan blue at 5 days after AAV vector exposure to HepG2. AAV‐DNase I did not induce cytotoxicity. *n* = 3. Data represent mean ± SD. The data were analyzed by two‐way ANOVA. Data represent mean ± SD. ****P < 0.0001.

The plasmid, pAAV‐ApoEHCR enhancer‐hAAT promoter‐hDNase I (hyperactive) ‐WPRE Xinact (AKA, pCLS‐014) was constructed as follows: The cDNA of the transgene expression cassette for CLS‐014 was synthesized by GenScript (Piscataway, NJ, USA). A 5′ EcoRI site and a 3′ HindIII site were added to the sequence of CLS‐014. The cassette consists of (a) apolipoprotein E‐hepatic control region (APOE‐HCR enhancer) and human alpha‐1‐antitrypsin (hAAT) promoter, (b) a Kozak sequence, (c) human hyperactive, actin resistant, DNase I‐variant containing the natural signal sequence, (d) a woodchuck hepatitis virus posttranscriptional element (WPRE) with the X protein coding region inactivated by mutating the start codon [WPRE‐X‐inactivated (woodchuck hepatitis virus posttranscriptional regulatory element with X protein inactivated)] (Fig. 1A). The CLS‐014 cDNA was digested with EcoRI‐HF and HindIII‐HF (New England Biolabs, Ipswich, MA, USA). An AAV promoterless expression vector, pAAV‐MCS‐Promoterless (which provides a polyA signal (pA) and two flanking AAV2 inverted terminal repeats, ITRs, for the CLS‐014 cassette) was purchased from Cell Bio Labs (Cat no. VPK‐411, San Diego, CA, USA). The pAAV‐MCS Promoterless vector was digested with EcoRI‐HF and HindIII‐HF, and similarly digested pCLS‐014 cassettes were ligated for 1 h at room temperature in a 3 : 1 insert to vector molar ratio. The ligated construct was transformed into SURE electrocompetent cells (Agilent Technologies, Santa Clara, CA, USA) and plated on LB‐Agar‐ampicillin plates. Colonies were selected and minipreps were prepared for screening. First, a SmaI digest was performed for AAV2‐inverted terminal repeats (ITR) integrity to determine that the expected bands were present. Bacterial clones were selected when the expected bands were present in each of the SmaI digest and the EcoRI‐HF and HindIII‐HF digest. The pCLS‐014 AAV plasmid (Fig. 1A) was subjected to whole‐plasmid sequencing (MGH DNA core), and then, plasmid was purified using an endo‐free Gigaprep (Qiagen, Carlsbad, CA, USA) performed by Alta Biotech (Aurora, CO, USA). We also engineered a control AAV plasmid called AAV‐null. AAV‐null contained all components of pCLS‐014 AAV plasmid with the exception that it was devoid of the DNase I cDNA.

### 2.5 AAV production

AAV‐DNase I vector was produced using large‐scale polyethylenimine triple plasmid transfections of rep/cap plasmid encoding the Anc80L65 capsid [30], pCLS‐014, and adenovirus helper plasmids in near‐confluent monolayers of HEK293 cells with further purification on an iodixanol gradient at scale using traditional protocols. The purified AAV‐DNase I vector was reformulated in PBS supplemented with 35 mm NaCl and 0.001% Pluronic F68, and quantitative PCR revealed a titer of 1.0 × 0^13^ genome copies per mL (GC per mL). The purified AAV vector was analyzed by SDS/PAGE, with three bands of 60, 72, and 90 kDa were observed in a ratio of ~ 1 : 1 : 10, which corresponds to the VP1‐3 proteins. AAV‐null contained all the components of CLS‐014 transgene expression cassette, with the exception that it was devoid of DNase I cDNA.

### 2.6 Histology

Formalin‐fixed, paraffin‐embedded liver and tumor sections were stained with hematoxylin and eosin for assessment of liver and tumor histology.

### 2.7 Immunohistochemistry and immunofluorescence

For immunolabeling of tumor specimens, resected mouse tumors were embedded in formalin. Slides were deparaffinized and rehydrated two times in xylene for 5 min, two times in 100% EtOH for 5 min, two times 95% EtOH for 1 min then 5 min in running H_2_O. Antigen retrieval was performed by heat induction in antigen unmasking solution (Vector Laboratories, Burlingame, CA, USA) using a microwave. Endogenous peroxidases were quenched, and slides were blocked in 10% goat serum (Vector Laboratories) for 1 h at 25 °C. Slides were incubated in primary antibody against CD45 (1 : 500 overnight at 4 °C) (BD Pharmagin, San Diego, CA, USA), CD4, and CD8 (1 : 500 overnight at 4 °C) (Abcam, Cambridge, MA, USA). Following three washes in PBS, slides were incubated in secondary antibody 45 min at 25 °C. After ABC and DAB (Vector Laboratories), slides were counterstained with hematoxylin. Negative controls included secondary antibodies alone. Two expert pathologists blinded to the study groups determined the percentage of the cells that stained positive for the above‐mentioned antibodies in tumors. This was determined by counting the number of positive cells in 10 random areas at a magnification of ×400 under light microscopy.

Murine tumor specimens were frozen in Tissue‐Tek® Optimal Cutting Temperature compound (Genprice Inc., San Jose, CA, USA) and stored at −80 °C. Cryostat (8‐μm sections) was used for immunofluorescence staining. Tissue was fixed in 2% paraformaldehyde for 30 min at room temperature and incubated with antihistone H3 antibody (Abcam) at 1 : 50 dilution and anti‐mouse Ly6G antibody (BD Biosciences, San Jose, CA, USA) at 1 : 50 dilution overnight at 4 °C. Following three washes with PBS, slides were incubated with fluorescent‐conjugated secondary antibody (Thermo Fisher Scientific) for 1 h followed by Hoechst nuclear staining. Negative controls included antibody isotypes and secondary antibody alone. Imaging was performed using Olympus Fluoview 3000 microscope (Olympus Corporation, Shinjuku, Tokyo, Japan).

### 2.8 Western blot

Cell lysates from tumor tissues were run on a 15% Bis‐Tris gel then transferred to a 0.2‐mm nitrocellulose membrane. After blocking in 5% BSA, membranes were incubated overnight at 4 °C with primary antibodies for histone H3 (Abcam) and β‐actin (Cell Signaling Technology, Danvers, MA, USA). Proteins were visualized using IRDye® 800CW goat anti‐rabbit 926–32 211 (1 : 10 000; LI‐COR, Lincoln, NE, USA) secondary antibodies. Images were captured with the Odyssey system (LI‐COR). The protein was semi‐quantified through the image studio Ver 5.2 software (LI‐COR, Lincoln, NE, USA).

### 2.9 MPO‐DNA quantification

To identify NETs in mouse serum, a capture ELISA myeloperoxidase (MPO) associated with DNA was performed as previously described [31].

### 2.10 DNase I concentration

The DNase I concentration in cell culture medium and plasma samples was analyzed using a fluorescent probe [32], representing hairpin oligonucleotide labeled with fluorescent dye and quencher. The increase in fluorescence was monitored using Varioscan Flash plate reader (Thermo Scientific). Culture medium and plasma samples were diluted by 20‐fold with the reaction buffer. 0.25 µm concentration of florescent probe was used. Calibration curve for DNase I concentration estimation was obtained using serial dilution of pure DNase I (Pulmozyme®, Dornase alfa) (Genentech, South San Francisco, CA, USA) assuming its concentration is 1 mg·mL^−1^ according to supplier and Mw 31 kDa. DNase I concentration in samples was estimated using linear regression (Fig. S1).

### 2.11 Quantitative polymerase chain reaction

RNA was extracted from liver tissues and purified (RNeasy Kit; Qiagen, Germantown, MD, USA), and 1 μg of total RNA was reverse‐transcribed into complementary DNA (PrimeScript RT Master Mix; Takara, Mountain View, CA, USA). Expression levels were determined by real‐time quantitative PCR (ABI Technologies, Foster City, CA, USA). The relative expression of target genes was normalized by β‐actin expression as an internal control. β‐Actin was measured by using the 5′‐GCTCTTTTCCAGCCTTCCTT‐3′, forward and 5′‐TGATCCACATCTGCTGGAAG‐3′, reverse primers. DNase I was measured by using the 5′‐ATGCGGTACAC‐AGGGCTAATG‐3′, forward and 5′‐AAAAGTCCGAATGTTGAAGGCT‐3′, reverse primers.

### 2.12 Serum analysis

Serum alanine aminotransferase (ALT), aspartate aminotransferase (AST), alkaline phosphatase, and total bilirubin were measured using HesKa Dri‐Chem 4000 (Heska, Loveland, CO, USA).

### 2.13 Flow cytometry analysis

The tumors were minced with surgical scissors and dissociated in an enzymatic solution of collagenase D (1 mg·mL^−1^) (Roche, Mannheim, Germany) and DNase I (100 μg·mL^−1^) for 30 min at 37 °C in a water bath with continuous agitation. After this treatment, the enzymatic solution was inactivated by adding DMEM with 10% FBS and was immediately centrifuged at 400 ***g*** for 5 min at 4 °C. The cell suspension was filtered through a 70‐μm cell strainer, subjected to discontinuous Percoll gradient separation (GE Healthcare, Pittsburgh, PA, USA). The cells were collected from their respective density fractions and prepared for flow cytometry analysis. One negative sample (no antibody) was used for gating purposes. Cell populations were determined by electronic gating based on forward versus side scatter. The CD45^+^ population was further characterized for T cells (CD3^+^/CD4^+^ and CD3^+^/CD8^+^) and neutrophils (CD11b^+^/Ly6G^+^). Flow cytometric data acquisition was performed using the LSRFortessa™ flow cytometer (BD Biosciences).

### 2.14 Statistical analysis

All values are expressed as the mean ± SD. Depending on the distribution (normal or non‐normal), either the *t*‐test, ANOVA, or the Mann–Whitney *U*‐test was performed in statistical analysis to compare differences between the groups. All statistical analyses were performed using graphprism 8 (GraphPad Software, Inc., La Jolla, CA, USA). The following designations apply to all figures: *P < 0.05, **P < 0.01, ***P < 0.001, ****P < 0.0001. *P‐*value < 0.05 was considered significant.

## 3 Results

### 3.1 AAV‐DNase I transduction of cultured human hepatoma cells leads to DNase I secretion

Human DNase I was cloned into an AAV expression plasmid (CLS‐014 AAV plasmid) with a liver‐specific promoter and packaged into the Anc80L65 capsid (Fig. 1A). HepG2 hepatoma cells were transduced with AAV‐DNase I to produce DNase I or AAV‐null as negative control. Cell culture media were collected over time to determine the levels of active DNase I. As shown in Fig. 1B, AAV‐DNase I efficiently transduced HepG2 cells and significantly increased DNase I concentration in cell supernatants continually up to 72 h post‐transfection. Similarly, DNase I concentration in the culture supernatant was also clearly increased in a dose‐dependent manner (1 × 10^4^–1 × 10^6^ GC/cell). The potential toxicity of AAV‐DNase I was compared with that of AAV‐null transduced cells (Fig. 1C). After AAV‐DNase I transduction, the proportion of dead cells in HepG2 cell culture at 5 days did not differ significantly with that of the AAV‐null transduced cells at any MOI examined. These findings indicate that AAV‐mediated DNase I gene transfer into cells of hepatocyte origin in vitro lead to sustainable secretion of DNase I in a dose‐dependent manner.

### 3.2 **Systemic administration of AAV‐DNase I mediates liver expression and secretion of DNase I** *in vivo*

**Fig. 2:**
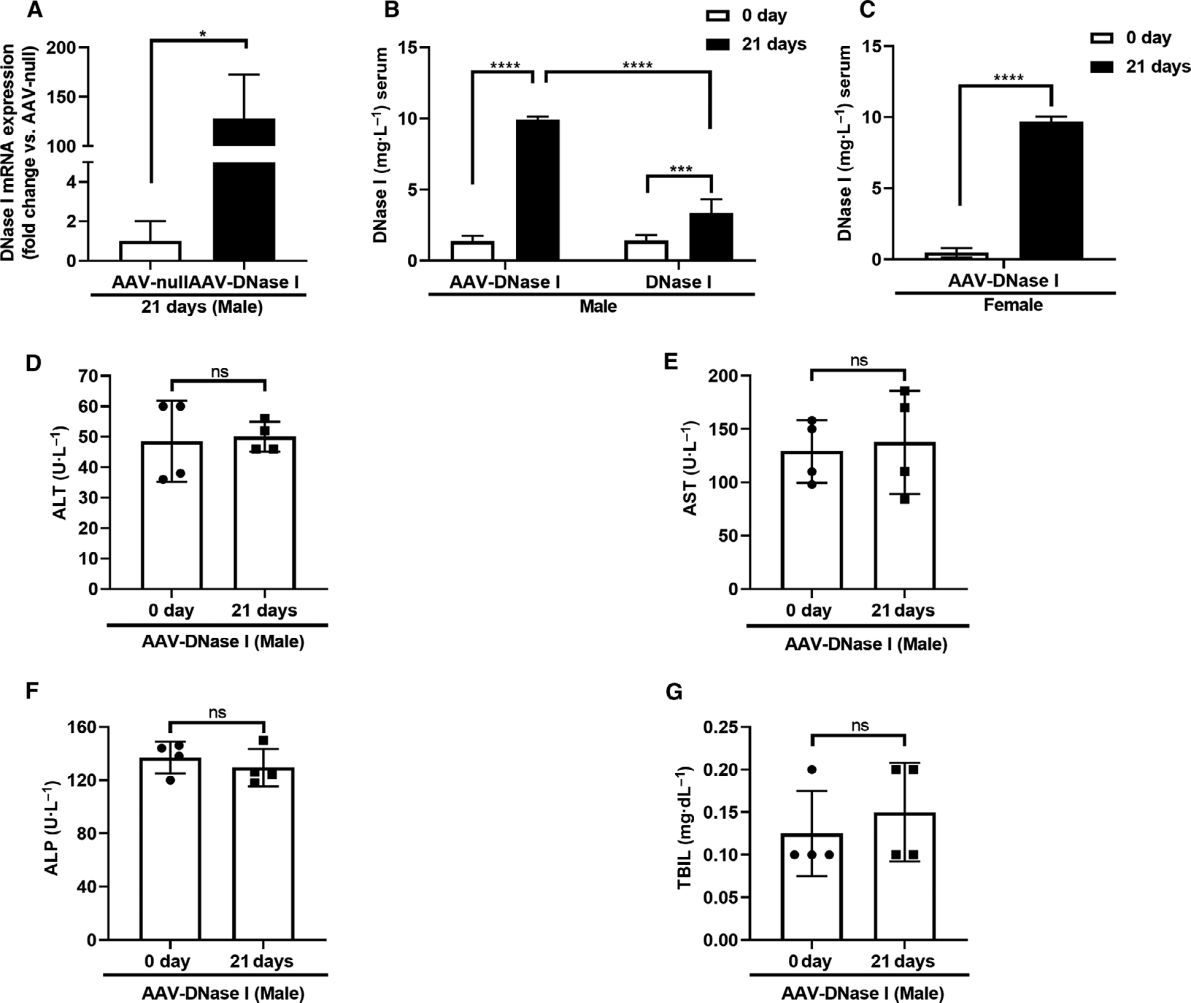
Systemic administration of AAV‐DNase I mediates liver expression and secretion of DNase I *in vivo*. (A) Gene expression of DNase I in AAV vector‐injected liver. Livers were isolated at 21 days from AAV‐DNase I group and total RNA was extracted. *n* = 3 mice/group. Data were analyzed by Welch's *t*‐test. (B, C) DNase I secretion from AAV‐DNase I‐treated mice after MC38 injection. AAV‐DNase I was systemically administered through the tail vein 4 days after MC38 injection. On day 17 after AAV‐DNase treatment, the levels of DNase I were significantly higher than levels before AAV‐DNase I injection and daily DNase I IP‐injected group in tumor‐bearing mice. *n* = 5 mice/group. Data were analyzed by two‐way ANOVA and Student's *t*‐test. (D, E, F, and G) The levels of hepatocellular injury and cholestasis markers (ALT, AST, ALP, TBIL). AAV‐DNase I did not increase markers of liver injury in wild‐type mice. *n* = 4 mice/group. Data were analyzed by Student's *t*‐test. All data represent mean ± SD. *P < 0.05, ***P < 0.001, ****P < 0.0001.

We next determined that a single IV AAV‐DNase I injection at 1.05 × 10^12^ GC per mouse resulted in significant increase of DNase I gene expression by day 21 in the liver, as compared with the AAV‐null group (*P* < 0.05, Fig. 2A). To compare the concentration of serum DNase I in the group of animals receiving single IV 1.05 × 10^12^ GC/mouse of AAV‐DNase I to animals receiving daily DNase I IP injections (50 μg·per mouse per·day), blood samples were assayed at day 0 and 21 after MC38 colon adenocarcinoma cells were injected via the portal vein. A dose of 1.05 × 10^12^ GC/mouse vector genomes was systemically administered through the tail vein 4 days after MC38 injection. Serum DNase I concentration was higher at day 17 after AAV‐DNase I treatment compared to mice that underwent daily DNase I injection via the IP route (*P* < 0.0001; Fig. 2B). Due to gender differences in AAV‐mediated transgene expression, we also detected DNase I concentration in female mice. The level of serum DNase I significantly increased after AAV‐DNase I injection in female mice at day 21 (Fig. 2C). To examine potential liver injury of AAV‐DNase I, markers of hepatocellular injury and cholestasis (ALT, AST, ALP, and total bilirubin) were evaluated at baseline and day 21 after AAV‐DNase I injection in wild‐type mice. We found no difference in hepatocellular injury and cholestasis markers in mice with and without AAV‐DNase I injection (Fig. 2D–G). These results suggest DNase I can be effectively expressed and released *in vivo* following AAV‐mediated DNase I liver gene transfer.

### 3.3 AAV‐mediated DNase I liver gene transfer reduces the growth of colorectal liver metastases

**Fig. 3:**
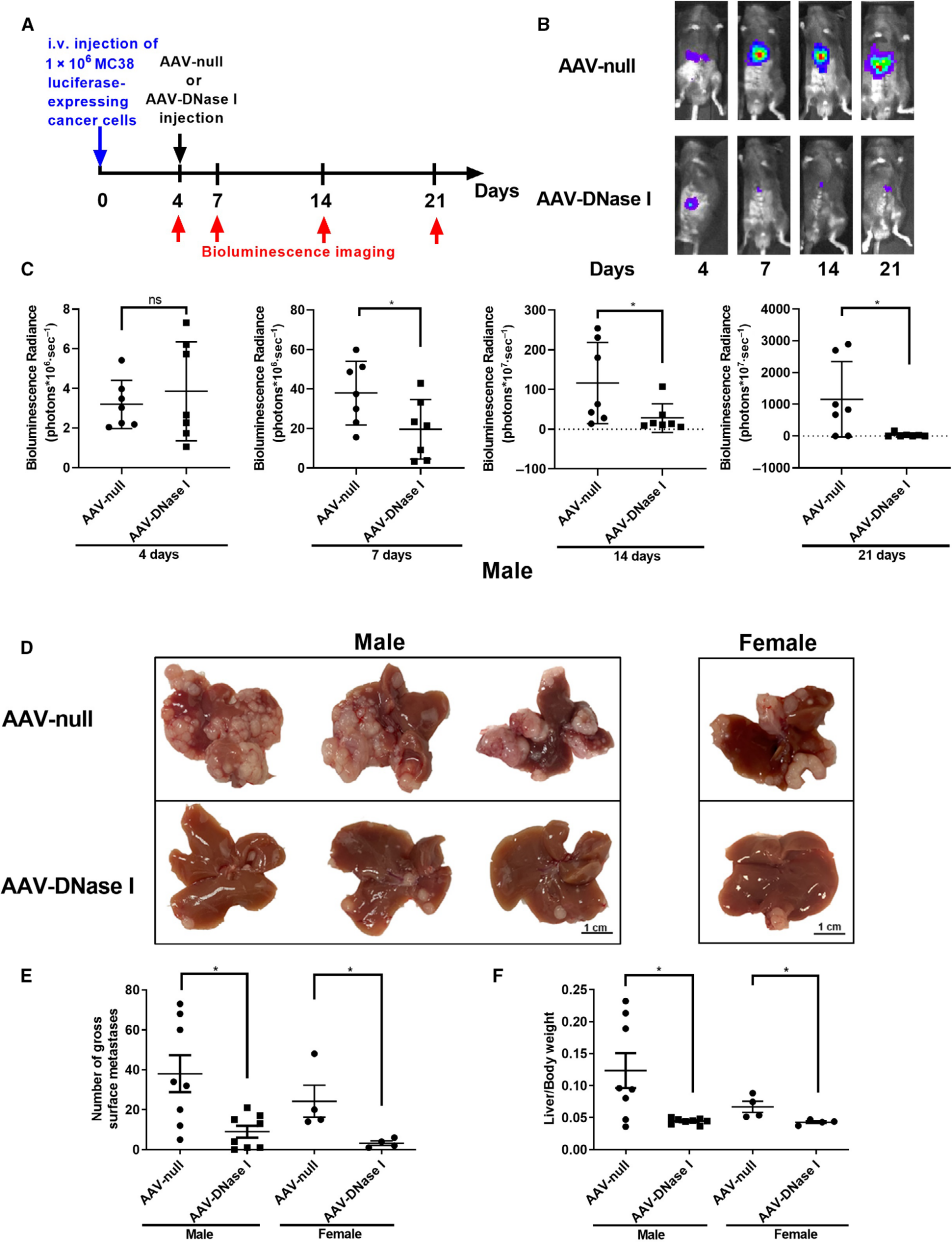
AAV‐mediated DNase I liver gene transfer reduces the growth of colorectal liver metastases. (A) Schematic showing experimental design *in vivo*. (B, C) The use of luciferase‐labeled MC38 cells allowed *in vivo* tracking of tumor growth with bioluminescence imaging. In the AAV‐DNase I‐treated group, we demonstrated that tumor growth is significantly inhibited at days 7, 14, and 21. *n* = 7 mice/group. Data were analyzed by Student's *t*‐test or Mann–Whitney *U*‐test. (D) Liver metastasis model using MC38 cells (1 × 10^6^) injected through the portal vein, showing smaller tumors harvested 3 weeks postinoculation in AAV‐DNase I‐treated mice compared with AAV‐null control. Scale bar = 1cm. (E, F) Graphs showing that AAV‐DNase I significantly decreased surface tumor nodules on liver and lower ratio of liver weight/body weight compared with AAV‐null group. *n* = 8 mice/group. Data were analyzed by Mann–Whitney *U*‐test. Data represent mean ± SD, *P < 0.05.

To determine whether AAV‐DNase I injection could inhibit the progression of CRC liver metastasis, we injected a luciferase‐expressing colon cancer cell line (MC38) into mice via the portal vein. Four days after MC38 cell injection, mice were given a single IV injection of 1.05 × 10^12^ GC/mouse of AAV‐DNase I or 1.05 × 10^12^ GC/mouse of AAV‐null as negative control and monitored for 21 days (Fig. 3A). Bioluminescent imaging shows metastasis development in AAV‐DNase I‐treated mice was lessened compared to mice in the AAV‐null group (*P* < 0.05; Fig. 3B,C). Previous work from our laboratory demonstrated that the development of gross metastases was attenuated by chronic administration of DNase I protein as it cleaves the DNA strands comprising NET structures [22, 23]. Similarly, in this study, single AAV‐DNase I administration inhibited the progression of liver metastasis in both male and female mice compared to the AAV‐null‐treated mice (Fig. 3D). AAV‐DNase I treatment also significantly decreased the number of metastatic nodules (*P* < 0.05) (Fig. 3E). A lower liver weight to body weight ratio in AAV‐DNase I‐treated mice was observed compared to AAV‐null treated mice (*P* < 0.05; Fig. 3F). These results further validate that AAV‐DNase I treatment inhibits the progression of liver metastasis in CRC model.

### 3.4 AAV‐mediated DNase I reduces neutrophil recruitment and inhibits NET formation in CRC liver metastasis and in circulation

**Fig. 4:**
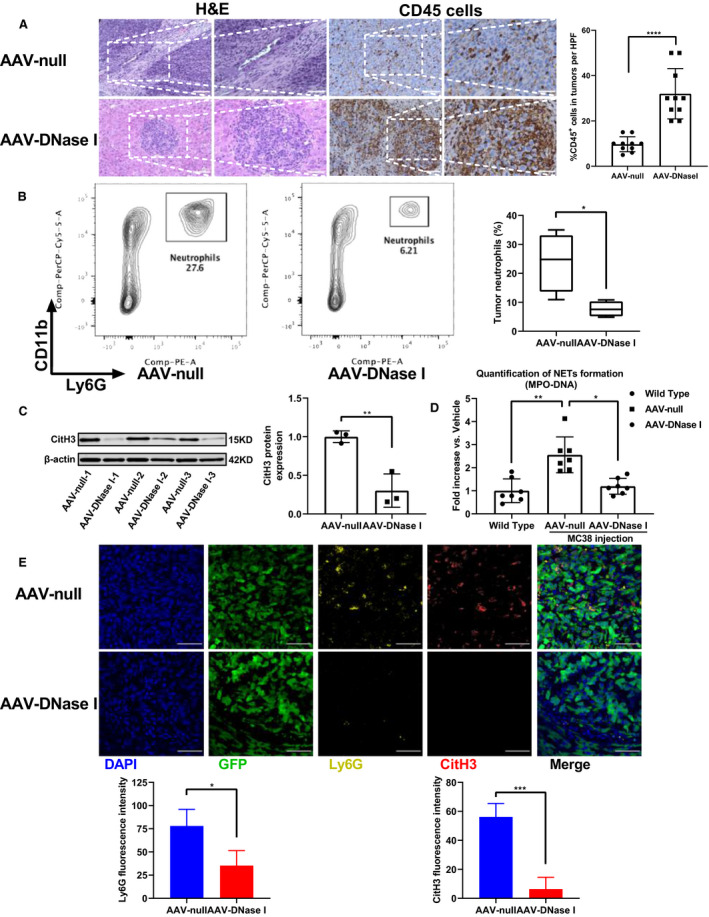
AAV‐mediated DNase I reduces neutrophil recruitment and inhibits NET formation in CRC liver metastasis and in circulation. (A) Representative images of H&E staining and immunohistochemical labeling for CD45 in the section exhibit increased immune cells in mice with AAV‐DNase I treatment compared with AAV‐null treated group. Quantification of CD45‐positive staining is expressed as a percentage average in the 10 fields. Magnification, ×200; magnified, ×400. Scale bar = 50 μm. *n* = 4 mice/group. Data were analyzed by Mann–Whitney *U*‐test. (B) Flow cytometry of tumor tissue showing neutrophils infiltration. *n* = 4 mice/group. Data were analyzed by Student's *t*‐test. (C and D) The expression of CitH3 by western blot analysis and quantification of serum MPO‐DNA by ELISA were assessed at 21 days. *n* = 3–7 mice/group. Data were analyzed by Student's *t*‐test and Kruskal–Wallis test. (E) Immunofluorescent staining of tumor tissue shows NET formation 21 days after tumor cell injection in AAV‐DNase I and AAV‐null treated mice. Nucleus, blue; tumor cells labeled with GFP, green; Ly6G, yellow; CitH3, red. Magnification, ×60. Scale bar = 50μm. Quantification of Ly6G positive or CitH3‐positive fluorescence intensity. *n* = 4 mice/group. Data were analyzed by Student's *t*‐test. All data represent mean ± SD, *P < 0.05, **P < 0.01, ***P < 0.001, ****P < 0.0001.

In the tumor microenvironment, the presence of immune cells plays a crucial role in cancer development [33]. HE staining revealed the tumor immune cell load following AAV‐null and AAV‐DNase I treatment at day 21. Based on previous studies, most of the immune cells present were CD45^+^ [34]. The percentage of CD45^+^ immune cell infiltration was significantly higher in AAV‐DNase I‐treated mice than the AAV‐null‐treated group (Fig. 4A; *P* < 0.0001). In human tumors, neutrophils make up a significant portion of the immune infiltrate in a wide variety of cancer types and increased levels of neutrophils and NETs in tumors is associated with poor prognosis in many patients with advanced cancers [13, 35]. Flow cytometry of tumor tissues revealed that neutrophil infiltration (CD11b^+^/Ly6G^+^ neutrophils) was significantly reduced in the AAV‐DNase I‐treated group (*P* < 0.05; Fig. 4B). Activated neutrophils can release NETs and facilitate capture of circulating cancer cells and support tumor metastasis [11]. In addition, disseminating tumor cells can in turn stimulate neutrophils to form NETs and treatment with NET‐digesting DNase I inhibits metastasis [36]. We thus examined whether the targets of DNase I could be tumor‐derived NETs in blood plasma since high levels of NETs and decreased DNase activity of blood plasma have been found in patients with metastatic cancers [37, 38]. The protein level of citrullinated histone H3 (CitH3), a marker of NET formation, was markedly lower in the tumors of AAV‐DNase I‐treated mice compared to AAV‐null‐treated mice (*P* < 0.01; Fig. 4C). Similarly, levels of NETs in the circulation as measured by MPO‐DNA complexes were significantly reduced in the AAV‐DNase I‐treated mice (*P* < 0.01, *P* < 0.05, Fig. 4D). Tumor tissues from both AAV‐DNase I and AAV‐null group were then harvested after 3 weeks of growth and stained for neutrophils (Ly6G), NETs (CitH3), and GFP‐labeled tumor cells. Immunofluorescent images revealed NETs were abundant in the AAV‐null treated mice but absent in AAV‐DNase I‐treated mice (Fig. 4E, *P* < 0.01, *P* < 0.001). These findings suggest that AAV‐mediated DNase I liver gene transfer inhibited neutrophil infiltration and NET formation in tumor tissues.

### 3.5 AAV‐mediated DNase I liver gene transfer recruits CD8^+^ T cells to CRC liver metastasis

**Fig. 5:**
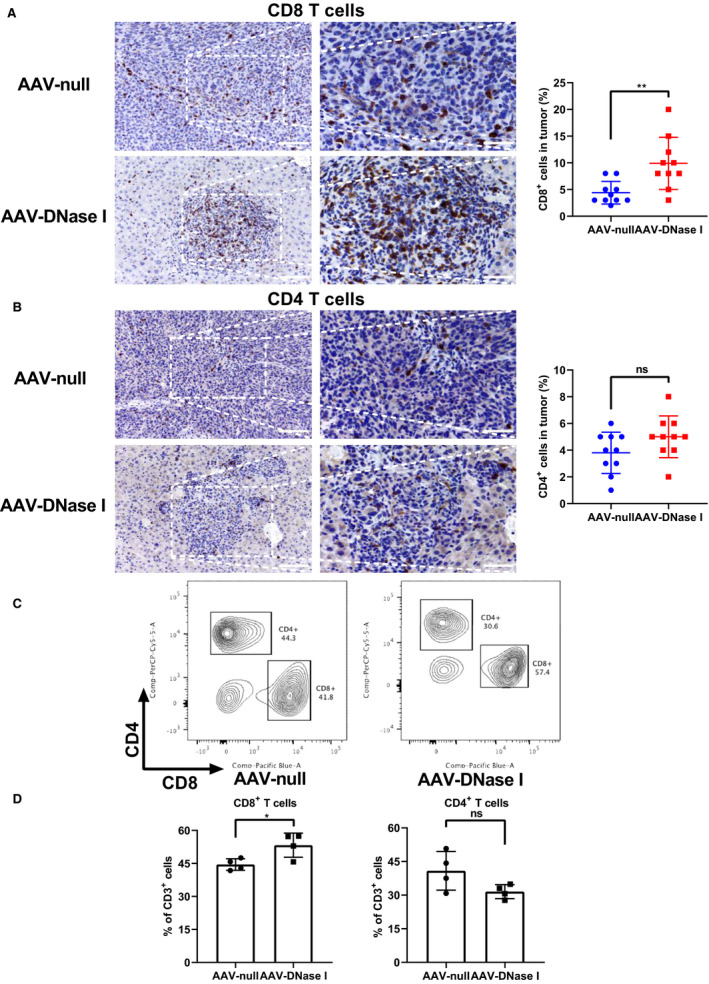
AAV‐mediated DNase I liver gene transfer recruits CD8^+^ T cells to CRC liver metastasis. (A and B) The ratios of CD8^+^ and CD4^+^ T cells in tumors. Quantification of CD4‐positive or CD8‐positive staining is expressed as a percentage average in the 10 fields. Magnification, ×200; magnified, ×400. Scale bar = 50 μm. Data were analyzed by Student's *t*‐test or Mann–Whitney *U*‐test. (C, D) Quantification of cellular percentages in tumor tissues by flow cytometry. Data were analyzed by Student's *t*‐test. All data represent mean ± SD; *n* = 4 mice/group, *P < 0.05, **P < 0.01.

Our results above demonstrate that AAV‐mediated DNase I liver gene transfer can modulate innate immune responses to the tumor. However, adaptive immune cells in the tumor microenvironment play an equally crucial role in tumor control. We next analyzed CD4^+^ T cells and CD8^+^ T cells in the metastatic tissue by immunohistochemistry. Compared to the AAV‐null treated group, the percentage of CD8^+^ T cells at the tumor site was greatly increased in the AAV‐DNase I‐treated group (*P* < 0.01, Fig. 5A). CD8^+^ T cells can detect and kill tumors through cytotoxic molecules, such as granzymes and perforin [39]. In contrast, the percentage of CD4^+^ T cells at the tumor sites in the AAV‐DNase I‐treated mice was not significantly different from those in the AAV‐null treated group (Fig. 5B). Similar results of the contrasting percentages of CD4^+^ and CD8^+^ T cells in tumor tissues were also detected and confirmed by flow cytometry (Fig. 5C,D). The proportion of CD8^+^ T cells was significantly higher in AAV‐DNase I‐treated mice when compared with AAV‐null treated mice (*P* < 0.05). Though not significant, the proportion of CD4^+^ T cells had a decreasing trend in AAV‐DNase I‐treated mice. These results suggest AAV‐mediated DNase I liver gene transfer induced antitumor immune responses through modulation of both innate and adaptive immune mechanisms.

## 4 Discussion

DNase I has been shown to be a potential biomarker in cancer patients as levels increase after successful treatment interventions and during remission. In addition, the failure of DNase I levels to increase in response to treatment is correlated with poor cancer prognosis [40, 41]. DNase I displays certain antimetastatic effects in a number of different tumor models [19, 20, 21, 22]. However, DNase I as a cancer therapeutic is questionable due to its short half‐life, potential requirement for lifelong use, and presence of potent DNase I inhibitors in the blood of cancer patients [42]. For example, actin, a major constituent of the microfilament system of eukaryotic cells can bind DNase I with nanomolar affinity and inhibit DNase I enzymatic activity almost entirely. Upon apoptotic and necrotic cell death, a significant amount of monomeric actin is released into the circulation thereby inhibiting DNase I catalytic activity in blood [43]. Methods to address this limitation in clinical use include studies utilizing DNase I‐coated nanoparticles [36]. Mice treated with DNase I‐coated nanoparticles displayed an increased concentration of DNase I but still required daily IP injection to reduce tumor metastasis. Similarly, DNase I attached to polysialic acid (PSA) has also been explored to improve *in vivo* pharmacological properties [44]. Although PSA could extend circulation time of DNase I by diminishing its excretion, its specific expression of DNase I was transient.

The present work provides evidence of inhibition of colorectal liver metastasis upon administration of an AAV vector encoding DNase I (CLS014). Since the majority of intestinal mesenteric drainage makes its first pass through the hepatic portal venous system, we consider liver‐specific expression with subsequent secretion of DNase I to liver sinusoids would be preferable delivery solution for CRC. We used a hepatotropic AAV utilizing Anc80L65 capsid to deliver DNase I into mouse hepatocytes and achieved a sustained DNase I expression *in vivo*. Anc80L65, a novel AAV capsid designed from in silico reconstruction of the viral evolutionary lineage, previously demonstrated robust transduction capabilities after local delivery into the liver compared with conventional AAVs [30]. Interestingly, the generation of NETs by neutrophils is accompanied by massive degradation of actin in the cytoplasm of neutrophils and release of actin into circulation [26]. The DNase I sequence in CLS014 transgene expression cassette has been reengineered to produce DNase I precursor with amino acid substitutions Q9R/E13R/N74K/A114F yielding DNase I protein with increased enzymatic activity and insensitive to actin inhibition [45, 46].

In this study, we use intraportal injection of colon cancer cells (MC38) to mimic the vascular spread of CRC metastasis. Compared with the commonly used injection of tumor cells into the spleen with subsequent splenectomy, intraportal injection is technically more difficult [47]. However, intraportal injection of tumor cells mimics the natural metastatic process more closely than intrasplenic, and splenectomy is not fit for the research of immune system. Although a rate of 10% portal venous thrombosis following intrahepatic injection is described [48], we did not observe any thrombotic complication when we injected one million MC38 cells in our models.

In this study, we achieved out translational objective: a single IV injection of CLS014 at 1.05 × 10^12^ GC dose was sufficient to reduce NET deposition in tumor tissue, to decrease levels of circulating NETs in blood, and to suppress the development of CRC liver metastasis in a preclinical immunocompetent mouse model without any signs of potential liver injury as measured by the end of treatment period. Fortunately, AAV vectors have been utilized in many clinical trials to deliver agents and have proven their safety and therapeutic efficacy in various diseases, including cancers [49]. AAV‐mediated DNase I liver gene transfer thus might provide efficient alternative to long‐term multiple daily injections of DNase I protein which otherwise might be required. However, NETs that are expelled from dying neutrophils are an important first‐line defense mechanism against bacterial, viral, fungal, and parasitic infections. Lifelong DNase I overexpression with digestion of NETs may have the potential to attenuate the control of pathogens. In addition, the recent study utilizing a different AAV9 variant and transgene indicates that systemic and sensory neuron toxicity may be general properties of IV delivery of AAV vectors at high doses, irrespective of the capsid serotype or transgene [50]. These potential negative effects require further investigation.

The tumor microenvironment is a complex network in which immune cells play pivotal roles in initiating and promoting cancer development or immune‐mediated tumor regression. The present work provides the evidence that AAV‐mediated DNase I liver gene transfer modulates both innate and adaptive immune responses in tumor microenvironment through suppression of neutrophil recruitment and parallel increase of tumor CD8^+^ T‐cell load. Neutrophils are the most abundant effector cells of innate immunity, and accumulating evidence has revealed the prominent role of neutrophils in infiltrating tumor tissues to promote growth, invasion, angiogenesis, and metastasis in various types of cancers [51]. Previous evidence suggests that release of NETs mediates the procancerous behavior of neutrophils [52]. Our results show that AAV‐mediated DNase I liver gene transfer significantly decreases the percentage of neutrophils and NET formation in metastatic tissue. In addition to physical cleavage of NET deposits, DNase I can reduce neutrophil infiltration and NET formation by attenuation TLR9 signaling [22], CXCL2 activation [53], and downregulation of CXCL2 and integrin α M expression [54].

Within the tumor microenvironment, the adaptive immune system is also highly involved in tumor control [55]. CD8^+^ T cells, as major effectors for antigen‐specific antitumor immunity, can recognize and kill malignant cells [56]. CD8^+^ T‐cell infiltration in liver metastasis is associated with better prognosis [57]. Our results indicate that AAV‐mediated DNase I liver gene transfer enhanced CD8^+^ T‐cell infiltration of liver metastasis. Interestingly, though not significant, the proportion of CD4^+^ T cells had a decreasing trend in AAV‐DNase I‐treated mice. It has been shown that recruitment of regulatory CD4^+^ T cells into tumors via secretion of CCL17 by tumor accumulating neutrophils represent a potent mechanism of impairment of local antitumor immunity [58]. It has been shown that NETs wrap and coat tumor cells and shield them from cytotoxicity, as mediated by CD8^+^ T cells and natural killer cells, by obstructing contact between immune cells and the surrounding target cells. Thus, reduced deposition of NETs might unlock the target access for immune effector cells. In addition, CD8^+^ T‐cell transwell migration was profoundly inhibited by NETs, while DNase I‐mediated removal of NETs restored CD8^+^ T‐cell migration [59]. The mechanism by which AAV‐DNase I induces CD8^+^ T‐cell infiltration is unknown. One possibility may be through DNase I treatment to increase sensing by TLR9 on CD8^+^ T cells. It has been demonstrated that TLR9 agonist effectively increases tumor infiltration by CD8^+^ T cells [60]. Furthermore, tumor accumulating neutrophils can produce NETs enriched with PD‐L1 thereby inhibiting cytokine production and proliferative capacity of tumor infiltrating lymphocytes through PDL‐1/PD‐1 axis. DNase I treatment reverses the functionality of tumor infiltrating lymphocytes.

Therefore, our findings suggest that AAV‐mediated DNase I liver gene transfer might rebalance innate and adaptive immune response in the tumor microenvironment and restore immune control on cancer cells as one of the mechanisms inhibiting CRC metastasis development in the liver.

## 5 Conclusions

Our research provides early evidence that AAV‐mediated DNase I liver gene transfer inhibits the development of CRC liver metastasis in preclinical setting and warrants further translational development of CLS014 gene therapy. Our findings highlight new perspectives for understanding the mechanisms that restore innate and adaptive immune response in the tumor microenvironment by targeting tumor‐associated NETs with AAV‐DNase I.

## Conflict of interest

CAM has a financial interest in Chameleon Biosciences, Inc., a company developing an enveloped AAV vector platform technology. CAM interests were reviewed and are managed by Massachusetts General Hospital and Partners HealthCare in accordance with their conflict of interest policies. CAM serves as a consultant of the scientific advisory board of CLS therapeutics, a gene therapy company with interest in developing an AAV‐based gene therapy for pancreatic cancer. GT and DG are co‐inventors on United States Patent Application 20190241908 for Treatment of diseases by liver expression of an enzyme which has a deoxyribonuclease (DNase) activity.

## Author contributions

AT and YX performed the conception and design; YX, JH, HZ, HW, AS, ST, AO, and VU executed the experiments; JH and YW acquired data; CAM, GT, VT, and DG involved in the development of methodology. YX, AT, GT, HH, and DG wrote, reviewed, and revised the manuscript.

## Supporting information


**Fig S1.** Calibration curve for DNase I concentration. (A) The typical calibration curve for 0.006‐100 mg·L^−1^ DNase I standards. (B) The linear range of calibration curve (0.01‐1 mg·L^−1^ DNase I standards). (C) Typical calibration curve for 0.006‐100 mg·L^−1^ DNase I standards, diluted in DMEM + 10% FBS. The sample without FBS could be used to estimate the background activity of the culture medium. (D) The linear part of fluorescence growth is used for the calculation of reaction rates.

## Data Availability

The raw research data are available from the corresponding author upon reasonable request.
