# Supplementary figures and images for: AAV‐mediated gene transfer of DNase I in the liver of mice with colorectal cancer reduces liver metastasis and restores local innate and adaptive immune response
Source: Mol Oncol. 2020 Sep 5;14(11):2920–35. doi: 10.1002/1878-0261.12787 (PMC7607180; doi:10.1002/1878-0261.12787)

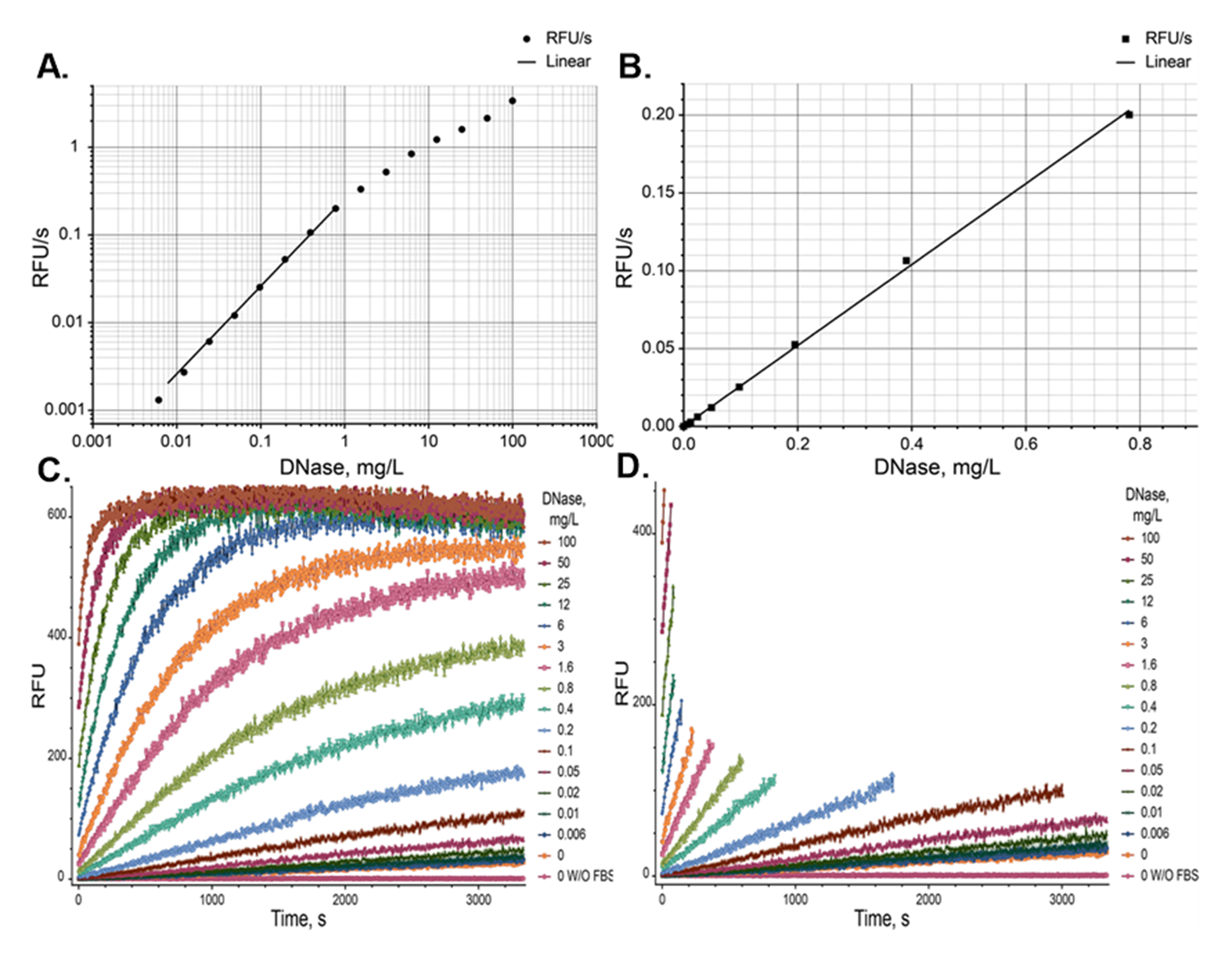

Supplement: Supplementary file 1 — Fig S1. Calibration curve for DNase I concentration. (A) The typical calibration curve for 0.006‐100 mg·L−1 DNase I standards. (B) The linear range of calibration curve (0.01‐1 mg·L−1 DNase I standards). (C) Typical calibration curve for 0.006‐100 mg·L−1 DNase I standards, diluted in DMEM + 10% FBS. The sample without FBS could be used to estimate the background activity of the culture medium. (D) The linear part of fluorescence growth is used for the calculation of reaction rates. [file MOL2-14-2920-s001.tif]
